# Exploring Yeast as a Study Model of Pantothenate Kinase-Associated Neurodegeneration and for the Identification of Therapeutic Compounds

**DOI:** 10.3390/ijms22010293

**Published:** 2020-12-30

**Authors:** Camilla Ceccatelli Berti, Alexandru Ionut Gilea, Marco Armando De Gregorio, Paola Goffrini

**Affiliations:** Department of Chemistry, Life Science and Environmental Sustainability, University of Parma, 43124 Parma, Italy; camilla.ceccatelliberti@unipr.it (C.C.B.); alexandruionut.gilea@studenti.unipr.it (A.I.G.); marcoarmando.degregorio@gmail.com (M.A.D.G.)

**Keywords:** pantothenate kinase, yeast, mitochondria, drug screening

## Abstract

Mutations in the pantothenate kinase 2 gene (*PANK2*) are the cause of pantothenate kinase-associated neurodegeneration (PKAN), the most common form of neurodegeneration with brain iron accumulation. Although different disease models have been created to investigate the pathogenic mechanism of PKAN, the cascade of molecular events resulting from CoA synthesis impairment is not completely understood. Moreover, for PKAN disease, only symptomatic treatments are available. Despite the lack of a neural system, *Saccharomyces cerevisiae* has been successfully used to decipher molecular mechanisms of many human disorders including neurodegenerative diseases as well as iron-related disorders. To gain insights into the molecular basis of PKAN, a yeast model of this disease was developed: a yeast strain with the unique gene encoding pantothenate kinase *CAB1* deleted, and expressing a pathological variant of this enzyme. A detailed functional characterization demonstrated that this model recapitulates the main phenotypes associated with human disease: mitochondrial dysfunction, altered lipid metabolism, iron overload, and oxidative damage suggesting that the yeast model could represent a tool to provide information on pathophysiology of PKAN. Taking advantage of the impaired oxidative growth of this mutant strain, a screening for molecules able to rescue this phenotype was performed. Two molecules in particular were able to restore the multiple defects associated with PKAN deficiency and the rescue was not allele-specific. Furthermore, the construction and characterization of a set of mutant alleles, allowing a quick evaluation of the biochemical consequences of pantothenate kinase (PANK) protein variants could be a tool to predict genotype/phenotype correlation.

## 1. Introduction

Pantothenate kinase-associated neurodegeneration, PKAN (OMIM #234200), is the most common form of neurodegeneration with brain iron accumulation (NBIA), a group of rare progressive neurological disorders characterized by excess iron accumulation in the *globus pallidus* and, to a lesser degree, in the *substantia nigra* [1,2]. PKAN is classified in two forms: classic, characterized by early onset, and atypical, which occurs later and progresses more slowly than the classic one [3]. Pantothenate kinase (PANK) catalyzes the ATP-dependent phosphorylation of pantothenate (vitamin B5) to 4′-phosphopantothenate, the first and limiting step in the coenzyme A (CoA) biosynthetic pathway. In mammals, four isoforms of PANK (1, 2, 3, 4) have been identified but only mutations in the *PANK2* gene, encoding isoform 2, are associated with PKAN. PANK2 is ubiquitous but widely expressed in the brain [4] and is localized in the mitochondrial inner membrane space [5,6,7] and possibly in the nucleus whereas PANK1 and PANK3 are predominantly found in the cytosol [8]. PANK4 is catalytically inactive and an alternative function for this protein has been attempted [9].

Deficiency in CoA biosynthesis could lead to a wide range of metabolic alterations due to the fact that about 9% of known enzymes use CoA as an obligate cofactor and due to its involvement in different pathways including tricarboxylic acid cycle, fatty acid oxidation and synthesis and amino acid metabolism [10]. The view that the defect in CoA biosynthesis is at the center of the molecular mechanism of PKAN is supported by the discovery that mutations in CoA synthase, *COASY*, cause a similar neurodegenerative disease, CoPAN (COASY Protein-Associated Neurodegeneration) [11]. The exact etiology of PKAN is still incomplete; Kotzbauer et al., [7] first suggested that alterations of mitochondrial and lipid metabolism could have a pivotal role in the pathogenesis of PKAN and that iron accumulation seems to be a secondary event [12]. To investigate the pathogenic mechanism of PKAN, different disease models have been created but none show all the dysfunctions associated with the disease. The *Drosophila melanogaster fumble* model recapitulates only partially the human phenotype, showing reduced whole-body CoA and locomotor dysfunction [13]. The *pank2* down regulation in Zebrafish resulted in an abnormal head development with smaller eyes, perturbation of brain morphology and an altered vasculature structure [14]. In the *Pank2* KO mouse model, a severe neurological phenotype is observed only when the mouse was subjected to a ketogenic diet [15] although recent investigations on *globus pallidus* of *Pank2* KO mice showed an alteration of CoA metabolism and secondary iron homeostasis, in dopamine metabolism as well as perturbation in mitochondrial enzymes activities [12]. Finally, a *Pank1* and *Pank2* KO mouse model showing a locomotor dysfunction, CoA deficiency associated with reduction of brain heme levels and oxidative metabolism impairment have been also described [16]. In vitro studies using PKAN patient fibroblasts and cultured neurons derived from induced pluripotent stem cells (iPSCs) generated a lot of results which, when taken together, highlight an alteration of the oxidative status and severe mitochondrial dysfunctions including impaired energy production, iron-sulfur cluster (ISC) and heme biosynthesis with consequent impairment of iron homeostasis [17,18,19,20,21].

Currently for PKAN disease only symptomatic treatments are available [22]. Therapeutic approaches using panthethine [13,15], CoA [19], acetyl-4′-phosphopantetheine [23], fosmetpantotenate [24], pantothenate [21] and 4′-phosphopantetheine [12] have been proposed since a beneficial effect in different PKAN models has been reported. Moreover, the oral administration of deferiprone in PKAN patients showed a reduction of brain iron and a slower progression of disease [25].

Yeast has been successfully used as a model organism to decipher molecular mechanisms of several human disorders and in recent years has proven to be a useful tool in drug discovery, allowing quick evaluation of the ability to suppress pathological phenotypes associated with specific mutation by screening of molecules libraries [26,27,28]. To this end we constructed and characterized a yeast model of PKAN, namely a yeast strain with the unique pantothenate kinase encoding gene, *CAB1* deleted and expressing pathological variants of *PANK2*. This model recapitulates the main phenotypes associated with human disease: mitochondrial dysfunction, altered lipid metabolism, iron overload, and oxidative damage, thus representing a useful model to investigate disease pathogenesis and identify new therapeutic molecules candidates for PKAN. A screening of the Selleck-FDA approved chemical library was performed and two molecules, 5,7 dichloro-8 hydroxyquinoline (CQ_CL_) and nalidixic acid (nalH), were identified that are able to restore all the phenotypes associated with CoA deficiency. Moreover, the construction and characterization of a set of different *cab1* pathological mutants allowed us to demonstrate that the beneficial effect of nalidixic acid does not depend on the type of mutation and to validate the use of yeast as an in vivo model that is able to quickly test the biochemical effects of *PANK2* mutations.

## 2. Results

### 2.1. PANK2 cDNA Did Not Complement Δcab1 Lethal Phenotype

In *Saccharomyces cerevisiae* there is only one gene encoding PANK, namely *CAB1*. To study *PANK2* mutations in yeast, we express human *PANK2* cDNA in a mutant strain devoid of *CAB1*. Since *CAB1* is an essential gene, it was disrupted in a strain carrying the wild-type allele cloned in the centromeric vector pFL39. In this condition, cell viability can be maintained thanks to the functional copy of plasmid-borne *CAB1. PANK2* cDNA was cloned in the multicopy yeast expression vector pYEX-BX under the control of the strong promoter *CUP1* and the recombinant construct was inserted into a Δ*cab1* mutant. The pFL39*CAB1* vector was then counter-selected by plasmid shuffling on synthetic complete medium (SC) without tryptophan plates. No transformant cells were able to grow in this condition, indicating that the *PANK2* cDNA failed to complement the lethal phenotype of Δ*cab1*.

The lack of complementation of the Δ*cab1* by the *PANK2* cDNA can be ascribed to different localizations of human and yeast enzymes. Alternatively, although in the yeast in vitro assay a mitochondrial sub-localization for PANK2 protein has been proposed [5], the importing of PANK2 in the heterologous mitochondria could not take place in vivo.

### 2.2. Cellular Localization of Cab1 Protein

**Figure 1:**
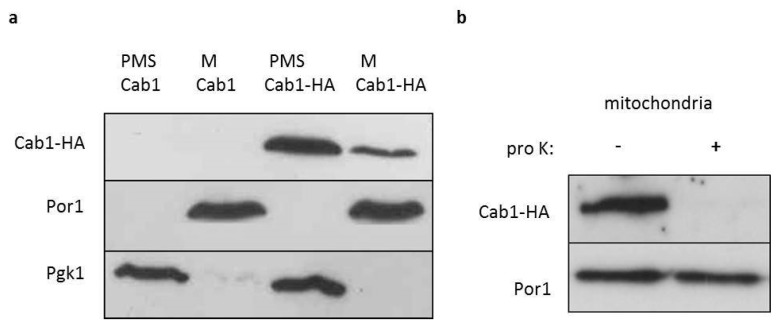
Localization of Cab1p. (**a**) Equal amounts (20 mg) of the mitochondrial fraction (M) and post-mitochondrial supernatant (PMS) extracted from Δ*cab1* strain harboring pFL39*CAB1* or pFL39*CAB1*HA were resolved by SDS-PAGE and analyzed by immunoblotting with HA, Pgk1 (cytosolic marker) and Por1 (mitochondrial outer membrane marker) antibodies. The experiment was performed on two independent clones for each strain (*N* = 1; *n* = 2) (**b**) Mitochondria were treated at 4 °C for 60 min with proteinase K (pro K) (1 mg/mL). The filter was incubated with anti-HA and anti-Por1 antibodies. The experiment was performed on two independent clones of Δ*cab1* strain harboring pFL39*CAB1*HA (*N* = 1; *n* = 2).

Although high-throughput studies suggest that Cab1 localizes in the cytoplasm and nucleus, an in silico analysis using the PSORT program [29] predicts both a cytosolic and mitochondrial localization of Cab1 protein. To investigate experimentally the Cab1p localization, equivalent amounts of mitochondrial pellet (M) and supernatant (PMS) fractions from cells expressing the wild-type or the HA-tagged wild-type *CAB1* allele were subjected to SDS-PAGE. As shown in Figure 1a the great majority of Cab1-HA co-fractionated with the soluble cytoplasmic protein phosphoglycerate kinase (Pgk1) while only a small amount remained with the mitochondrial membrane protein porin (Por1) in the mitochondria. To further investigate the sub-mitochondrial localization of this fraction of Cab1p, a protease protection assay of intact mitochondria was performed. After the treatment, no HA signal was detected indicating a vulnerable physical association of Cab1 with the outer mitochondrial membrane (Figure 1b) thus demonstrating that the PANK in yeast behaves predominantly as a cytoplasmic enzyme.

### 2.3. Construction of PKAN Yeast Model

More than 150 mutations in the *PANK2* gene that spread throughout the whole gene have been identified so far [30]. These can map onto the active site, dimerization interface and protein interior [31]. Seventy (70) of them are missense while the remaining are frameshift and nonsense mutations, stop codons, splicing errors or deletions/insertions.

PANK2 and yeast Cab1 proteins were first aligned to investigate the conservation of the mutated amino acid residues (Figure S1) and 29 missense mutations were found to affect conserved residues. On the basis of the crystallographic mapping of missense mutations previously carried out [31] and of the crystal structure of human PANK2 (PDB:5e26), four residues—D217, G219, V226, and G521—localized on the ATP-binding site; seven—E241, Y361, D378, Y383, N404, Y536, and G544—on the protein surface while the remaining on the dimerization domain or in the protein interior (Table S1).

To create a yeast model of PKAN, namely a strain with the *CAB1* deleted and expressing pathological variants of pantothenate kinase, we initially constructed two mutant alleles, one representative of the ATP-binding domain, *cab1*^G311R^ corresponding to *PANK2*^G521R^, and the other of the dimerization domain *cab1^N290I^* corresponding to *PANK2*^N500I^. To take into consideration the hypothesis that these mutations determine a complete loss of function, we also constructed a yeast strain expressing the thermosensitive allele *cab1^G351S^* previously described [32]. Single point mutations were introduced in pFL39*CAB1*-HA using mutagenic overlap PCR and the recombinant plasmids obtained were inserted in Δ*cab1* strain carrying a wild-type *CAB1* on a *URA3*-bearing vector (pFL38*CAB1*). The different transformant strains were then plated on 5-FOA medium to select for cells that have lost the pFL38*CAB1*. The strains expressing *cab1^N290I^* and *cab1^G351S^* mutants were able to grow, indicating that they are both hypomorphic alleles being able to complement the Δ*cab1* lethal phenotype. These mutant strains were further characterized. On the contrary, the strain expressing *cab1*^G311R^ did not support growth, representing a loss of a function allele.

### 2.4. Characterization of the PKAN Yeast Model

#### 2.4.1. Pantothenate Kinase Deficient Strains Exhibit Mitochondrial Dysfunction

**Figure 2:**
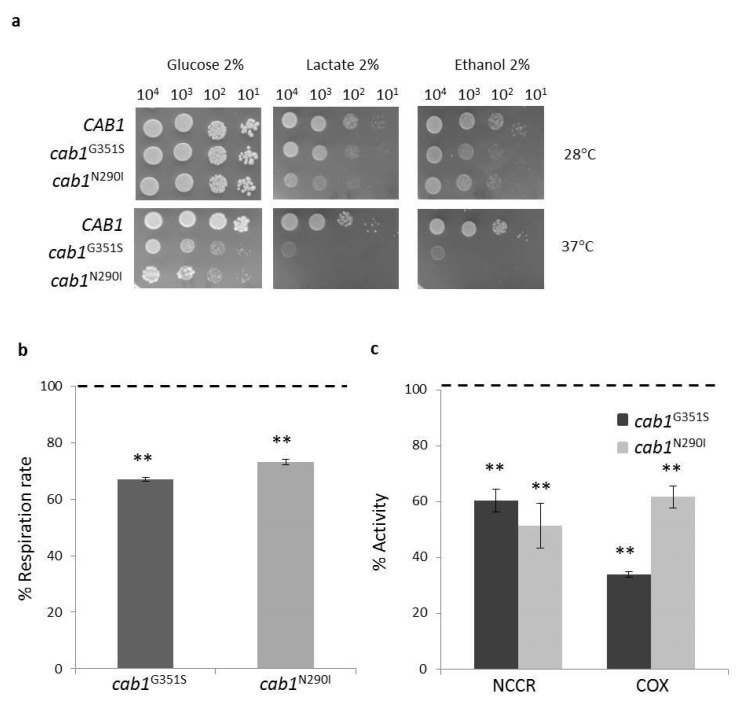
(**a**) Oxidative growth phenotype. Equal amounts of serial dilutions of cells from exponentially grown cultures (10^4^, 10^3^, 10^2^, 10^1^ cell/spot) were spotted onto SC medium supplemented with the indicated carbon. The growth was scored after 3–5 days of incubation at 28 °C and 37 °C. The experiment was performed on at least three independent clones for each strain. (*N* = 2; *n* = 6). (**b**) Oxygen consumption rate (OCR) was measured in wild-type and mutant strains. (**c**) NADH-cytochrome c oxidoreductase (NCCR) and cytochrome c oxidase (COX) activities were measured in mitochondria extracted as described in Material and Methods. OCR and mitochondrial enzyme activities were normalized to the strain transformed with the *CAB1* wild-type allele. Values are means ± standard deviation. *N* = 3; *n* = 9 for each strain. **: *p* < 0.01.

Coenzyme A is an important cofactor of the Krebs cycle and a defect in its biosynthesis leads to an alteration of cell energy metabolism. Moreover, we previously demonstrated an impairment in mitochondrial function in a yeast deficient of CoA synthase enzyme in which the level of CoA in mitochondria was reduced by 40% compared to wild-type [33]. To investigate mitochondrial function in the PKAN model, the ability of the two mutant strains to use respiratory vs fermentable carbon sources at 28 °C and 37 °C has been evaluated by spot assay analysis. Both mutant strains exhibited a clear growth defect on respiratory substrates both at 28 °C and 37 °C (Figure 2a). In addition, *cab1*^N290I^ showed a thermosensitive phenotype (ts) on glucose similar to what was observed for *cab1^G351S^* [32]. To better quantify the mitochondrial defect, the oxygen consumption rate (OCR) was measured. Results show that both *cab1^G351S^* and *cab1*^N290I^ mutant strains had a lower OCR of around 35% and 30% respectively compared to the wild-type (Figure 2b). Likewise, the NADH-cytochrome c oxidoreductase (NCCR) and cytochrome c oxidase (COX) activities were significantly reduced (Figure 2c). Although a measurement of CoA content has not been performed in these mutant strains these results indicate a mitochondrial dysfunction associated with a defect in the CoA biosynthetic pathway in our model system.

#### 2.4.2. Pantothenate Kinase Deficient Strains Show Iron Homeostasis Perturbation

**Figure 3:**
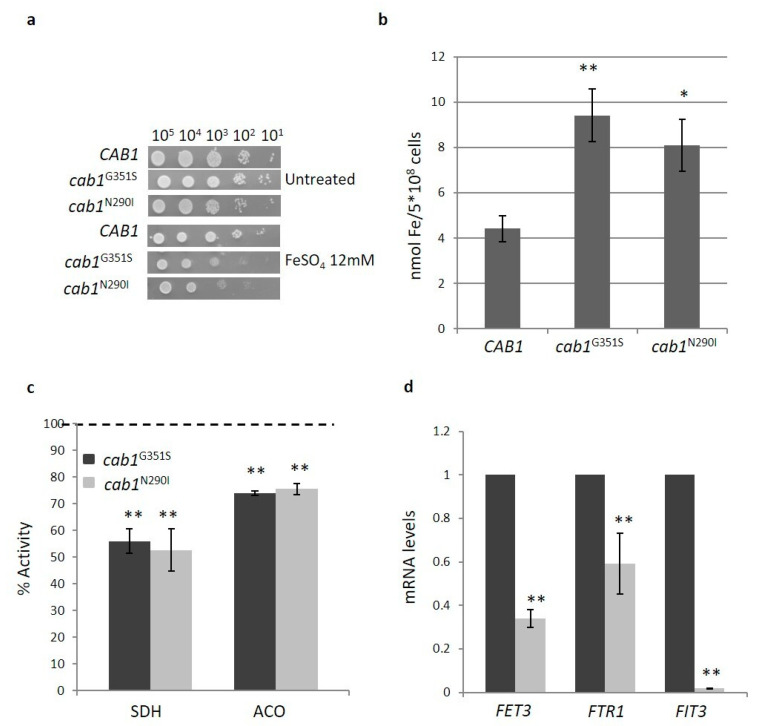
(**a**) Iron sensitivity. Equal amounts of serial dilutions of cells from exponentially grown cultures (10^5^, 10^4^, 10^3^, 10^2^, 10^1^ cell/spot) were spotted onto SC medium supplemented with 12 mM FeSO_4_. The growth was scored after 3 days of incubation at 28 °C. The experiment was performed on at least three independent clones for each strain (*N* = 2; *n* = 6). (**b**) Cellular iron content was quantified in cells grown to the early stationary phase in SC medium plus 2% galactose. Values are means ± standard deviation. *N* = 3; *n* = 12 for each strain. *: *p* < 0.05; **: *p* < 0.01. (**c**) Succinate dehydrogenase (SDH) and aconitase (ACO) activities were measured in mitochondrial-enriched fraction or whole-cell extracts as described in ‘Material and Methods’. The activities were normalized to the strain transformed with the *CAB1* wild-type allele. Values are means ± standard deviation. *N* = 3; *n* = 9 for each strain. **: *p* < 0.01. (**d**) The total RNA was extracted from wild-type and mutant strain grown in SC medium plus galactose 2% as described in ‘Materials Methods’. The mRNA levels of iron regulon genes were quantified by RT-qPCR. Values are reported as mRNA level normalized with respect to wild-type. Expression of *FET1*, *FTR1*, and *FIT3* was normalized to the mRNA levels of the internal control *ACT1*. Values are means ± standard deviation. *N* = 1; *n* = 9 each strain. **: *p* < 0.01.

PKAN disorder is characterized by iron deposition in the brain although the relationship between CoA deficiency and iron accumulation remains unclear [22]. To investigate if PANK deficiency perturbs iron homeostasis in our model, the cellular response to iron was evaluated. It has been previously reported that an excessive iron accumulation in the mitochondria led to an increased sensitivity to this ion [34,35] and for this reason the inhibition of the growth upon addition of FeSO_4_ to the medium was tested. As shown in Figure 3a, both mutant strains, *cab1^G351S^* and *cab1*^N290I^, show a decreased growth in the medium containing FeSO_4_. A quantitative determination of cellular iron level was then performed by a colorimetric assay [33]. As shown in Figure 3b both mutants exhibited a two-fold increase in iron content with respect to the parental strain. Considering the relationship between iron accumulation and biosynthesis of the cluster Fe-S (ISC), a marker of mitochondrial functionality linked to iron metabolism, the activity of the Fe-S enzymes, succinate dehydrogenase (SDH) and aconitase was then assayed. Both activities were significantly decreased with respect to the wild-type with SDH showing around 50% reduction and aconitase exhibiting a 25% reduction (Figure 3c).

It is known that *S. cerevisiae* responds to a reduced ISC synthesis by increasing the cellular iron uptake and by changing intracellular iron re-distribution [36]. To investigate if iron accumulation observed in our PKAN model was due to transcriptional activation of “iron regulon genes”, we used Real time PCR to analyze the expression level of three key genes: *FET3* (Ferro-O_2_ oxidoreductase*)*, *FTR1* (High affinity iron permease) and *FIT3* (Facilitator of Iron Transport). As depicted in Figure 3d, all three genes are down-regulated (0.60-, 0.40-, and 1-fold respectively), indicating that iron accumulation in PKAN yeast is not due to an increased uptake of this ion.

#### 2.4.3. Pantothenate Kinase Deficient Strains Show Altered Oxidative Status

**Figure 4:**
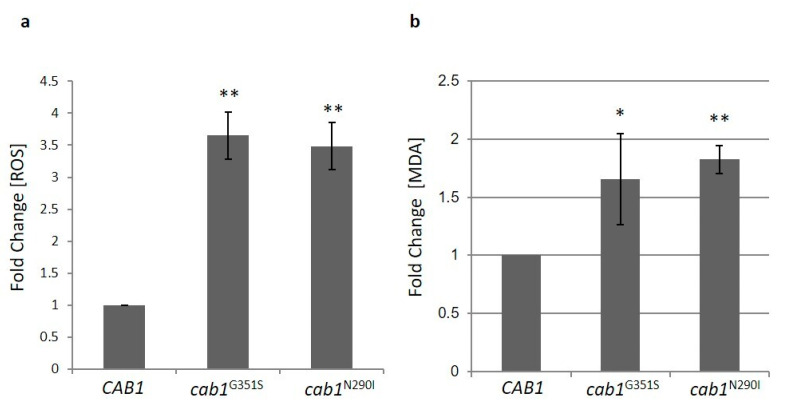
(**a**) ROS content evaluation by dichlorofluorescein diacetate assay in wild-type and mutant strains grown to the early stationary phase in SC medium containing 2% galactose. (**b**) Lipid peroxidation was evaluated by measuring malondialdehyde content in whole-cell extracts. The contents of ROS and MDA in *cab1* mutants were normalized to the strain transformed with the *CAB1* wild-type allele and the values are reported as fold change with respect to the wild-type. Values are means ± standard deviation. *N* = 4; *n* = 12 for each strain. *: *p* < 0.05; **: *p* < 0.01.

High iron content causes hypersensitivity to oxidative stress [35,37,38] and to reactive oxygen species (ROS)-inducing agents. In line with this both *cab1^G351S^* and *cab1*^N290I^ mutant strains exhibited increased sensitivity to H_2_O_2_ (unpublished results). The quantification of ROS was then performed using the dichlorofluorescein diacetate (DCFDA) assay as depicted in Figure 4a both mutant strains showed a 3.5-fold increase in ROS with respect to the parental strain.

High levels of ROS could impair membrane function through lipid damage. ROS attack lipids, such as polyunsaturated fatty acid (PUFAs), glycolipids, phospholipids and cholesterol [39] which gives rise to the lipid peroxidation process a chain reaction that produces multiple breakdown molecules such as malondialdehyde (MDA) and 4-hydroxynonenal (HNE). To assess lipid peroxidation, we measured the content of the specific biomarker MDA [40]. In both mutant strains a significant increase of MDA content was detected (Figure 4b).

#### 2.4.4. Pantothenate Kinase Deficient Strains Show Alteration in Lipid Metabolism

CoA acts as a carrier of the acyl group making it crucial for fatty acid synthesis (FAS) which plays many roles in the cells, from the production of sterols and membrane phospholipids to the generation of non-polar lipids, triacylglycerols and steryl esters that are stored in so-called lipid particles/droplets and serve as energy reserves [41].

**Figure 5:**
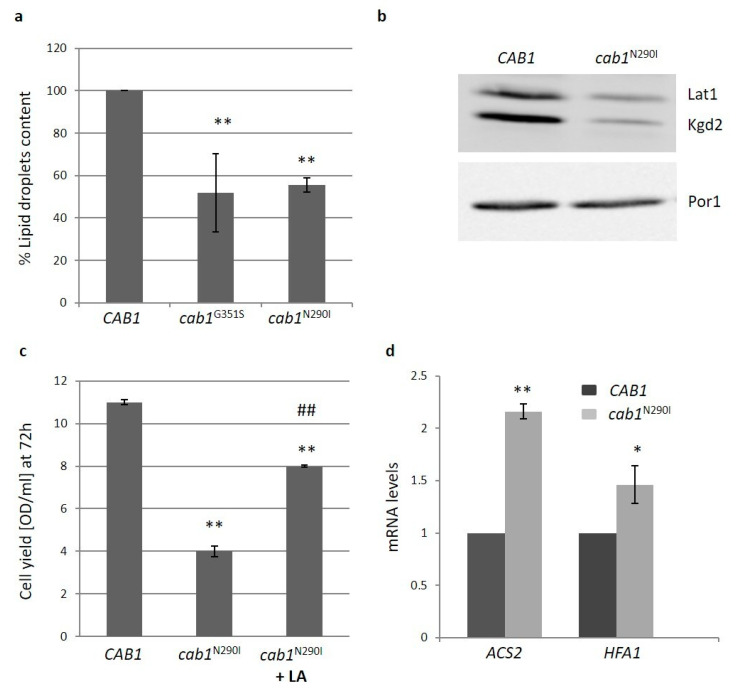
(**a**) The intracellular lipid droplets content was detected by fluorimetric analysis after incubation of wild-type and mutant strains with the fluorescent lipophilic dye Nile Red (4ug/mL). The values corresponding to *cab1* mutants were normalized to the strain transformed with the *CAB1* wild-type allele. Values are means ± standard deviation. *N* = 3; *n* = 9 for wild-type and *cab1*^G351S^ strains, *n* = 12 for *cab1*^N290I^ strain. **: *p* < 0.01. (**b**) Representative image of western blot analysis of lipoylated subunits of PDH (Lat1p) and α-KGDH (Kgd2) extracted from strain harboring pFL39*CAB1* or pFL39*cab1*^N290I^. *N* = 3; *n* = 3 for each strain. (**c**) Cell Yield (OD_600_/mL) was evaluated by growing wild-type and mutant strains in SC medium containing 2% ethanol for 72 h in the presence of 100 mM Lipoic Acid (LA). Values are means ± standard deviation. *N* = 1; *n* = 6 for each strain and condition. **: *p* < 0.01 relative to *CAB1* wild-type strain and ##: *p* < 0.01 relative to *cab1*^N290I^ mutant strain. (**d**) The total RNA was extracted from cells grown in SC medium plus glucose 0.6%. The mRNA level of *ACS2* and *HFA1* genes was quantified by RT-qPCR. Values are reported as mRNA level normalized respect to wild-type Expression of *ACS2* and *HFA1* was normalized to the mRNA levels of the internal control *ACT1.* Values are means ± standard deviation. *N* = 1; *n* = 9 for each strain. *: *p* < 0.05; **: *p* < 0.01.

As recently demonstrated, the deficit in PANK in yeast leads to different levels of sterol intermediates [42] and in a yeast model of CoPAN showing a 40% reduction of coenzyme A, the content of intracellular lipid droplets is reduced [33]. We then evaluated the content of intracellular lipid droplets in the PKAN yeast model. The fluorimetric analysis highlighted a reduction of lipid droplets of about 50% in the mutant strains (Figure 5a) suggesting that FAS is impaired when Cab1p activity is affected.

Acetyl-CoA availability is also crucial for mitochondrial FAS (mtFAS) which is necessary for synthesis of lipoic acid (LA), a sulfur-containing cofactor required for the function of several mitochondrial enzymes including pyruvate dehydrogenase (PDH) and α-ketoglutarate dehydrogenase (α-KGDH) [43]. Using an antibody that detects protein-bound LA we used Western Blot to analyze the levels of two lipoylated proteins: one of the PDH complex (α-Lat1) and the other of the α-KGDH complex (α-Kgd2). As shown in Figure 5b decreased amounts of both proteins were detected in the mutant *cab1*^N290I^. The ability of LA supplementation to partially restore the oxidative growth defect of the mutant strain indicates an impaired synthesis of lipoic acid in PKAN model (Figure 5c).

To better investigate lipid metabolism, the expression of two genes involved in fatty acid metabolism, *ACS2* (acetyl-CoA synthase) and *HFA1* (mitochondrial acetyl-coenzyme A carboxylase), was measured by Real time PCR. The expression of both genes was significantly increased (Figure 5d) suggesting that the mutant cells try to increase the synthesis of the fundamental precursor acetyl-CoA to counteract the alteration of lipid metabolism.

### 2.5. Search for Chemical Suppressors for Pantothenate Kinase Deficiency

**Figure 6:**
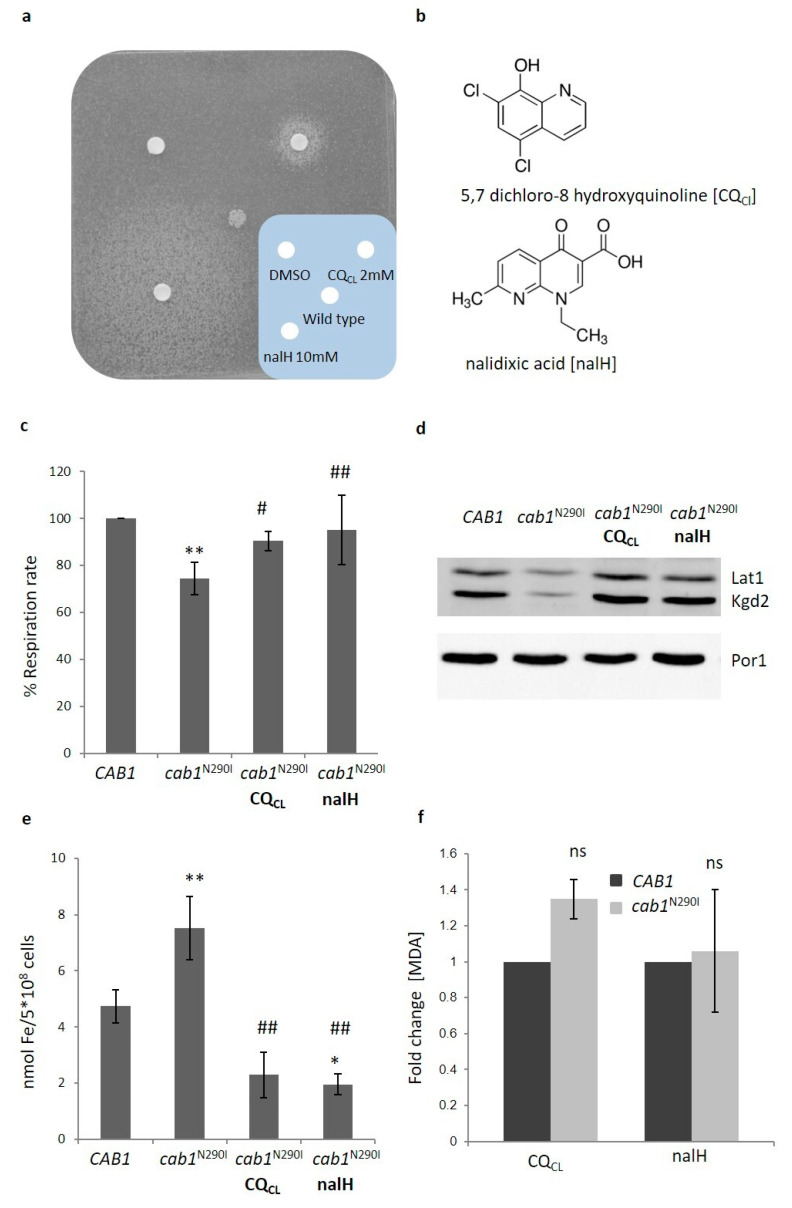
(**a**) 4 × 10^5^ (*cab1*^N290I^) cells were plated on YP medium supplemented with 2% ethanol. Three sterile filters were soaked with 2.5 µl of each compound or DMSO. Plates were incubated at 28 °C for 5 days. (**b**) Chemical structure of 5,7 dichloro-8 hydroxyquinoline (CQ_CL_) and nalidixic acid (nalH). (**c**) Oxygen consumption rate was evaluated in wild-type and mutant strains grown with or without CQ_CL_/nalH. OCR was normalized to the strain transformed with the *CAB1* wild-type allele. Values are means ± standard deviation. *N* = 3; *n* = 9 for each strain and condition. **: *p* < 0.01 relative to *CAB1* wild-type strain and #: *p* < 0.05; ##: *p* < 0.01 relative to *cab1*^N290I^ mutant strain. (**d**) Representative image of western blot analysis of lipoylated subunits of PDH (Lat1p) and α-KGDH (Kgd2) extracted from strain harboring pFL39*CAB1* or pFL39*cab1*^N290I^ grown with or without CQ_C_L/nalH. The filter was incubated with the specific antibody anti-Lipoic Acid and anti-Por1. *N* = 3; *n* = 3 for each strain and condition. (**e**) Cellular iron content was quantified in cells grown in the presence of CQ_CL_ or nalH. Values are means ± standard deviation. *N* = 4; *n* = 12 for each strain and condition. *: *p* < 0.05; **: *p* < 0.01 relative to *CAB1* wild-type strain and ##: *p* < 0.01 relative to *cab1*^N290I^ mutant strain. (**f**) MDA content was evaluated in wild-type and mutant strains grown with CQ_CL_/nalH. Values are means ± standard deviation. *N* = 1; *n* = 9 for each strain and condition. ns: not significant.

Although the therapeutic effect of exogenous CoA or 4′-phosphopantetehine administrations in reverting/correcting pathological phenotypes in PKAN-derived neurons or *PKAN* mouse model has been demonstrated [12,19] no established treatment for PANK2-linked diseases is available. The yeast PKAN model that recapitulates the most important phenotypes found in PKAN patients could represent a powerful tool for the searching of a chemical suppressor i.e., a potential therapeutic drug for PKAN. Taking advantage of the oxidative growth defect showed by mutant strains, a screening of the Selleck chemical library containing 1018 molecules authorized by the FDA for the treatment of several diseases, was performed. The molecules were tested by the drug drop test for their ability to suppress the respiratory growth defect of the *cab1^G351S^* mutant strain [44] after setting the best conditions (temperature, inoculum, respiratory substrates) for the screening. Eight molecules were classified as positive in both primary and secondary screenings and were called CRMs (*CAB1* Rescuing Molecule) (unpublished results). We then tested if these drugs were able to restore the growth defect of the *cab1*^N290I^ mutant strain that mimics the pathogenic mutation *PANK2*^N500I^. Among the four molecules that were also identified as active in this strain we decided to study in more detail the two compounds CRM1 and CRM4 that was the most effective; CRM1 is 5,7 dichloro-8 hydroxyquinoline (CQ_Cl_) and CRM4 is nalidixic acid (nalH), (Figure 6a). CQ_Cl_ is a heterocycle compound belonging to the quinoline family with 8-hydroxyquinoline (8HQ) being its precursor (Figure 6b). It has several bioactivities including antimicrobial, anticancer, anti-inflammatory, antioxidant and antineurodegenerative activity [45]. NalH is the first of the synthetic quinolone antibiotics, a large group of synthetic antibacterial based on a 4-oxo-1,8-naphthyridin-3-carboxylic acid nucleus and active against aerobic Gram-negative microorganisms (Figure 6b).

#### CQ_Cl_ and nalH Rescue All the Pathological Phenotypes of the PKAN Model

We have therefore evaluated the ability of both molecules to rescue the other phenotypes associated with CoA impairment. We first determined the CQ_CL_ and nalH optimal working concentration in a liquid ethanol medium by growing *cab1*^N290I^ mutant strain in the presence of different concentrations of each molecule. The growth defect in the presence of CQ_Cl_ was reduced as from the 0.0625 µM concentration and disappeared completely at 1 µM. nalH was active in the concentrations between 0.5 µM and 32 µM (Figure S2a,b). For further analyses we used CQ_Cl_ at a concentration of 1 µM and nalH at 4 µM. The respiratory activity as well as the activities of mitochondrial respiratory complexes (NCCR, SDH and COX) of PKAN model were rescued in the presence of the drugs (Figure 6c and Figure S3a). Moreover, the level of the lipoylated proteins Lat1 and Kgd2, returned to the wild-type level when the mutant strain was incubated with the molecules, suggesting that LA synthesis was at least partially restored (Figure 6d). Finally, the molecules rescued both iron and ROS content together with lipid peroxidation (Figure 6e,f and Figure S3b). Since a common feature of both molecules, CQ_CL_/nalH, is the ability to bind bivalent ions including iron [46,47], it could be hypothesized that the observed rescue is mainly due to the restoration of the physiological iron content.

### 2.6. Construction of a Set of cab1 Mutants to Assay PANK Activity and to Validate the Effectiveness of the Drugs

As previously mentioned, about 70 missense mutations associated with PKAN have been identified in *PANK2* gene [30] and in many cases their impact on protein function is not known. Moreover, the correlation between residual activity and the age of onset and/or the rate of progression of the disease, i.e., a possible genotype-phenotype relationship, has been marginally investigated and evidence of possible differences between in vivo and in vitro assays have been reported [48]. We therefore decided to construct other yeast strains expressing known missense mutations spanning different domains of the protein so as to investigate the in vivo biochemical consequences of these variants and evaluate the beneficial effect of the positive molecules previously found on a greater number of mutants. The advantage of this model is that *S. cerevisiae* possesses only one PANK gene and therefore substitutions leading to a complete loss of function are easily identified since they do not complement the Δ*cab1* lethal phenotype and mutations that partially compromise the PANK activity are able to complement the lethal phenotype. The further characterization of this category could allow, albeit indirectly, to correlate the residual activity to the severity of the phenotype. From the 13 new mutants constructed, regarding conserved residues between PANK2 and Cab1 proteins (Table S1), six were unable to grow, thus behaving as null allele, while the others were able to complement the null *cab1* lethal phenotype. The majority of these mutant strains, with the exception of *cab1*^N170I^ and *cab1*^D144G^, showed a reduction in oxidative growth at 37 °C, although to different extents (data not shown). In most mutants, OCR was affected between 40% and 80% of the wild-type. On the basis of the oxidative phenotype, we classified the mutations as: (1) null (mutant alleles unable to complement Δ*cab1*), (2) severe (for example *cab1*^S237N^), (3) mild (for example *cab*^I294V^) and wild-type (for example *cab1*^D144G^) (Table 1).

**Figure 7:**
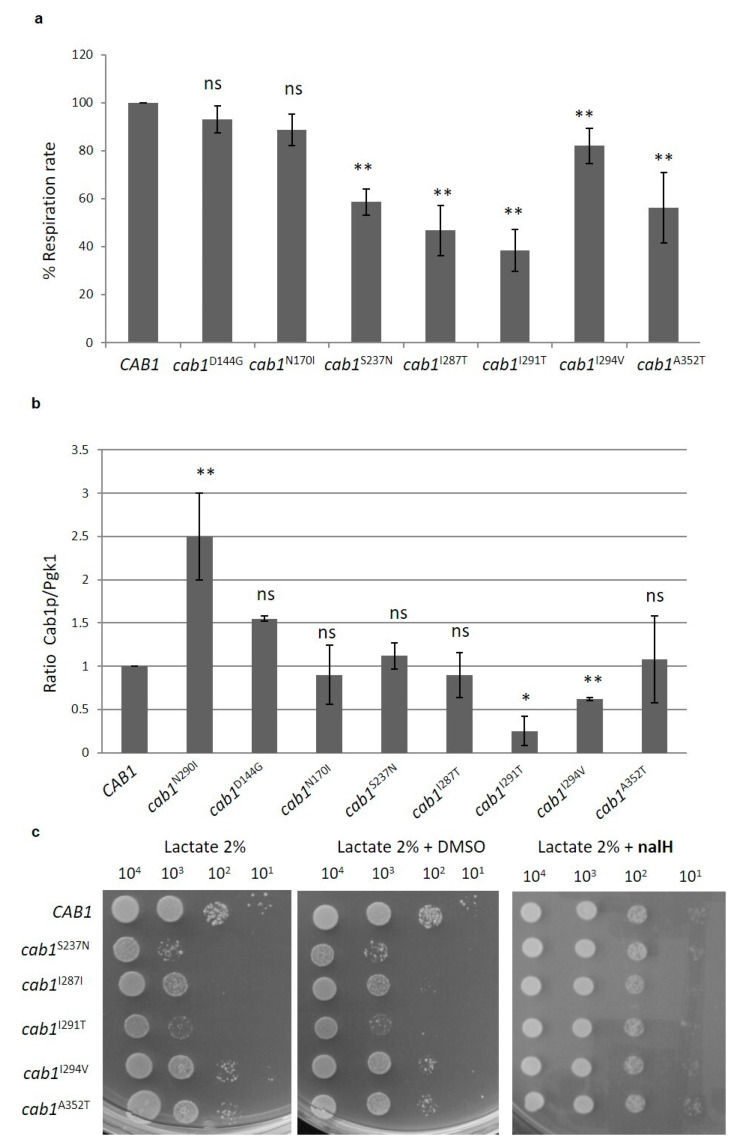
(**a**) OCR was measured in wild-type and mutant strains grown at 37 °C in SC medium supplemented with 0.6% glucose. Values are means ± standard deviation. The OCR was normalized to the strain transformed with the *CAB1* wild-type allele. Values are means ± standard deviation. *N* = 3; *n* = 9 for each strain. **: *p* < 0.01; ns: not significant. (**b**) Steady state level of Cab1 protein in wild-type and mutant strains. The proteins were extracted from wild-type and mutant strains grown at 37 °C, or 28 °C for *cab1*^N290I^, in SC medium supplement with 0.6% glucose. Quantification of protein bands was performed using the Image Lab software. *N* = 2; *n* = 2 for each strain. *: *p* < 0.05; **: *p* < 0.01; ns: not significant. (**c**) Equal amounts of serial dilutions of wild-type and mutant strains from exponentially grown cultures were spotted (10^4^, 10^3^, 10^2^, 10^1^ cell/spot) onto SC medium plates supplemented with 2% lactate, 2% lactate with DMSO and 2% lactate with 31 µM nalH. The growth was scored after 4 days of incubation at 37 °C. The experiment was performed on at least three independent clones for each strain (*N* = 2; *n* = 6).

To evaluate if the defects observed are caused by protein instability, we measured the steady state levels of Cab1 mutant proteins normalized to Pgk1. The protein levels were similar to that of the wild-type except for N290I which showed a 2-fold increase and for the I291T and I294V mutants for which 75% and 38% decreases was observed respectively (Figure 7b). Since in these two mutant strains the *cab1* gene expression is similar to that observed in the wild-type (unpublished results) we can speculate that the impaired phenotype observed in these mutants is caused by a reduced protein level. In the other cases the defects are associated with a deficiency of enzyme activity.

To assess if the nalH rescue could be dependent on the type of *cab1* mutation, we analyzed if the addition of this latter molecule to the growth medium was able to rescue the OXPHOS defect showed by the *cab1*^S237N^, *cab1*^I287T^, *cab1*^I291T^, *cab1*^I294V, and^ *cab1*^A352T^ mutant strains. To this aim a spot assay analysis was performed and, as shown in Figure 7c, in the presence of 31 µM nalH all mutant strains partially or totally recovered the OXPHOS defect, suggesting that the positive action of this molecules does not depend on the type of *cab1* mutation.

## 3. Discussion

The powerful genetics and molecular tools available, together with the acquired extensive knowledge of *S. cerevisiae*’s biology made this yeast a suitable model to elucidate the pathogenic mechanism of many human disorders. Despite the lack of a neural system, yeast has been successfully used for studying neurodegenerative diseases [26] as well as iron-related disorders [53]. PKAN is a severe and disabling neurodegenerative disorder associated with an inborn error of the CoA biosynthetic pathway and characterized by brain iron accumulation with pathogenic molecular mechanisms that have not been fully explained yet even if different PKAN disease models have been generated and characterized [54]. No effective treatment for this neurodegeneration is available.

In this study, we provide a detailed characterization of a yeast model of PKAN, i.e., a strain with the unique gene encoding PANK *CAB1* deleted and expressing a pathological hypomorphic variant of this enzyme that is able to support cell viability. This model recapitulates the main phenotypes found in patients thus representing a useful tool to investigate the pathogenic mechanism of this disease and to perform a screening of a chemical library to search for potential therapeutic drug for PKAN. The yeast PKAN model showed mitochondrial dysfunction: oxidative growth, OCR and the activity of respiratory chain complexes (NCCR, SDH and COX) were significantly reduced. Iron accumulates in the cells and the activity of succinate dehydrogenase and aconitase—enzyme markers of mitochondrial functionality linked to iron metabolism—is decreased. The high iron level could explain the increase in ROS content and the consequent activation of the lipid peroxidation process observed in the mutant, suggesting a membrane damage. The expression level of three key genes of yeast iron regulon—*FET3*, *FTR1*, and *FIT3—*is reduced, suggesting that iron accumulation is not due to an increased uptake of this ion but, rather, due to the iron not being correctly used in ISC biosynthesis possibly accumulating and the cells down-regulating the genes involved in iron uptake to counteract this accumulation. Accordingly, in *PANK2* knockout cell lines a significant increase in ferroportin (*FPN*) and in mitochondrial ferritin (*FTMT*) expression was observed, enhancing iron export and iron mitochondrial storage [20]. Moreover, the PKAN mouse model senses and responds to an increase in cytosolic iron by decreasing the expression of Tfrc (transferrin receptor 1) and Ireb2 (iron regulatory protein 2) [12].

Considering that acetyl-CoA, a CoA derivative, is the primary substrate for cytosolic and mitochondrial FAS, we investigated both these pathways. A reduction of lipid droplets and of the steady state level of lipoylated subunit of PDH complex (α–Lat1) and of α-KGDH complex (α–Kgd2) was observed in our model indicating that lipid metabolism is affected. The finding that the addition of LA to the growth medium partially rescue the respiratory growth defect of the mutant strain supports the hypothesis that the synthesis of LA is reduced. To counteract the acetyl-CoA deficiency, the cells respond by up-regulating the expression of two genes involved in fatty acid biosynthetic process, *ACS2* and *HFA1*.

Although in the PKAN yeast model a reduction of the CoA content can only be hypothesized based on the analysis of the mutated variants of PANK enzyme and by analogy with what previously demonstrated in the CoPAN yeast, we can speculate that a decrease in the CoA level could impair mtFAS and consequently LA synthesis thus reducing the activity of lipoylated enzymes of the Krebs cycle, PDH and α-KGDH. Defects in Krebs cycle is associated with a reduced production of GTP and NADH resulting in an impairment in mitochondrial ISC biogenesis, loss of activity of Fe-S-dependent enzymes and oxidative phosphorylation [18,55]. These effects together with the alteration of membrane composition and permeability could represent a primary mechanism which triggers abnormal intracellular iron content generating oxidative stress and lipid peroxidation. According to Mena et al., [56] mitochondrial dysfunction, iron accumulation, and oxidative damage generate a feedback loop of increased iron accumulation and oxidative stress. The same hypothesis was recently made by other authors to explain the cascade of molecular events resulting from loss of PANK 2 [12,57].

The screening of the Selleck chemical library, composed of 1018 compounds, allowed the identification of two molecules—5,7 dichloro-8 hydroxyquinoline (CQ_CL_) and nalidixic acid (nalH)—which were found to be able to rescue the respiratory growth defect of yeast PKAN model. Both molecules were also able to revert the multiple phenotypes associated with defects in PANK activity.

CQ_CL_ belongs to the quinoline family and it has been demonstrated that 8-hydroxyquinoline (8HQ), its precursor, can restore metal homeostasis in models of Alzheimer’s disease [47]. Furthermore, besides its antimicrobial activity nalH is also able to complex with iron and has been found to be able to reduce the sensitivity to the ROS inducing agent menadione in a yeast model of Hailey-Hailey disease [58]. On the basis of these properties, we can speculate that the mechanism that underlies their rescue ability could lie on their property to chelate the excess iron and reducing the oxidative stress and maybe the membrane damage.

However, the mechanism underlying the ability of the two molecules to recover the multiple defects of the mutant may not be the same. CQ_CL_ but not nalH is also effective in restoring oxidative growth of a strain expressing a pathological allele of CoA synthase (unpublished results). Other experiments are needed to elucidate how each molecule acts on the different phenotypes of PKAN yeast. Furthermore, we demonstrated that the positive effect exerted by nalH is not allele-specific since this compound rescued the oxidative growth defect of different *cab1* pathological mutants provided that there is a residual enzyme activity left—supplementation of these compound in 5-FOA medium does not support cell viability of null mutant alleles.

Another poorly investigated aspect is the correlation between the residual activity of the enzyme and the age of onset and/or the rate of progression of the disease, i.e., a possible genotype-phenotype relationship. In fact, the biochemical consequences of the known missense mutations have only been partially studied. To this aim the construction and characterization of a set of *cab1* pathological mutants allowed us to quickly test the effect of 13 *PANK2* mutations. The lack of complementation of the ∆*cab1* lethal phenotype by a mutant allele indicates a loss of function allele while the ability to complement the null ∆*cab1* indicates a hypomorphic allele. All the mutants, except two, showed a respiratory growth defect and an impairment of OCR, thought at different extent. The steady state level of the different mutated proteins also allowed us to speculate if the affected phenotype could be ascribed to protein instability or to a reduced enzyme activity. The proposed classification of the mutations based on the results obtained—null, severe, mild, and wild-type—seems to correlate with the severity of the pathology and with the age of onset (Table 1).

Overall, our data established yeast as an appropriate in vivo model to evaluate the pathogenicity of *PANK2* mutations and to help clarify the cascade of molecular events consequent to pantothenate kinase deficiency. Moreover, this model represents a useful tool for high-throughput screening of potential effective molecules for disorders linked to inborn error of CoA biosynthesis which must be validated in more complex cellular and animal models.

## 4. Materials and Methods

### 4.1. Yeast Strains and Media

Yeast strains derived from W303-1B (Matα *ade2-1 leu2-3, 112 ura3-1 trp1-1 his3-11, 15 can1-100*) [59] were grown in SC media (0.69% yeast nitrogen base without amino acids) (Formedium™, Hunstanton, UK) supplemented with 1 g/L drop-out mix (DO) except for the amino acids and bases needed to retain plasmids. Various carbon sources were added at 2% (*w/v*) (Carlo Erba, Milan, Italy) in liquid phase or after solidification with 20 g/L agar (Formedium™, Hunstanton, UK). 1 mg/mL 5-fluoroorotic acid (5-FOA) monohydrate (Formedium™, Hunstanton, UK) was supplemented into SC medium. YP medium contains 0.5% Yeast extract, 1% Peptone (Formedium™, Hunstanton, UK)) and YPA medium was YP 2X supplemented with 40 mg/mL adenine base (Formedium™, Hunstanton, UK).

### 4.2. Plasmid and Mutant Strains Construction

The plasmids used in this work are the centromeric vectors pFL38 or pFL39 that include *URA3* and *TRP1* respectively as selection markers [60] and the multicopy expression vector pYEXBX (Clontech, Mountain View, CA, USA, *URA3* marker). The *CAB1* gene and its upstream and downstream regulatory regions were PCR amplified using genomic DNA of strain W303-1B as template and CAB1Fw and CAB1Rv as primers and cloned in both pFL38 and pFL39 thus obtaining pFL38*CAB1* and pFL39*CAB1.* HA-tag was inserted at the 3′ end of *CAB1* allele through the PCR overlap extension method [61] with the specified primers (Supplementary Table S1). *PANK2* cDNA (kindly supplied by Dr. V. Tiranti, Istitito Neurologico Besta, Milan, Italy) was cloned in the *Bam*HI and *Sal*I digested pYEX plasmid. The plasmid pFL39*CAB1* was then introduced into the W303-1B strain through the Li-Ac method [62], and one-step gene disruption [63] of *CAB1* gene was performed in this strain since the loss of *CAB1* is lethal. The KanMX4 cassette flanked by *CAB1* sequences was amplified from plasmid pFA6a plasmid [64]. Transformation and selection on YPD (YP containing 2% glucose) medium supplemented with 200 mg/mL geneticin (Formedium™, Hunstanton, UK) allowed to obtain W303-1B Δ*cab1*/pFL39*CAB1*. Gene disruption of *CAB1* to a chromosomal locus was confirmed by PCR.

The *cab1* mutant alleles were obtained by site-direct mutagenesis of *CAB1*-HA-tagged gene using the overlap extension technique [61] and appropriate primers (Supplementary Table S2). The mutated alleles were then cloned into the pFL39 vector and verified by sequencing. To obtain the mutant strains, the Δ*cab1*/pFL38*CAB1* was transformed with pFL39cab1* mutant alleles and the pFL38*CAB1* was lost through plasmid shuffling as previously reported [63]. Restriction-enzyme digestions, *Escherichia coli* transformation, and plasmid extractions were performed with standard methods [65].

### 4.3. Oxidative Growth, Respiratory Activity, and Mitochondrial Enzymes Assays

For oxidative growth analysis, strains were serially diluted and spotted on SC-W agar plates, supplemented with 2% ethanol, 2% lactate or 2% glucose as control. Plates were incubated at both 28 °C and 37 °C. The OCR (Oxygen consumption Rate) was measured at 30 °C for whole cells cultured for 18 h at 28 °C in SC medium supplemented with 0.6% glucose until exhaustion using a Clark-type oxygen electrode (Oxygraph System, Hansatech Instruments, King’s Lynn, UK) with 1 mL of air-saturated respiration buffer (0.1 M phthalate–KOH, pH 5.0) and 10 mM glucose. The reaction started by the addition of 20 mg of wet-weight cells as described [66]. For mitochondrial enzyme activities, measurement cells were grown to mid-logarithmic phase in SC medium supplemented with 0.2% glucose and 2% galactose. Complex II (SDH), NADH-cytochrome c oxidoreductase (NCCR) and complex IV (COX) specific activities were measured spectrophotometrically on a mitochondrial-enriched fraction as previously described [33,67,68]. Aconitase activity was measured in whole-cell extracts and assayed by the aconitase-isocitrate dehydrogenase-coupled assay [35]. Quantification of protein concentration was performed by the method of Bradford [69] using Bio-Rad protein assay following the manufacturer’s instructions.

### 4.4. Measurements of Iron Content, ROS Content, Lipid Peroxidation, and Lipid Droplets

The iron content was determined by a colorimetric assay as previously described [33] from cells grown to late-log phase in SC medium supplemented with 0,2% glucose and 2% galactose. ROS quantification was determined using dichlorofluorescein diacetate (DCFDA) that inside the cells can be oxidized by ROS to dichlorofluorescein (DCF), according to Marchetti et al. [70] with minor modifications. 1 × 10^7^ cells grown until the stationary phase were incubated with DCFDA (20 µM) (Sigma-Aldrich^®^, Darmstadt, Germany) for 30 min at 28 °C, washed twice with PBS (phosphate buffered saline), and resuspended in lysis buffer (20 mM Tris HCl pH 7.5, 150 mM NaCl, 1 mM EDTA, 1% Triton X-100 and 2.5 mM Na_4_P_2_O_7_). The samples were broken down with glass beads for 3–4 times for 30 s and 100 µl of whole-cell extract was collected in a new tube. The fluorescence measurement by the “Tecan SPECTRAR Fluor Plus” system (Tecan, Männedorf, Switzerland) (excitation at 485 nm and emission at 535 nm) indirectly indicates the amount of ROS. The fold change in ROS content in mutant strains to that of wild-type was reported. The lipid peroxidation was evaluated by measuring malondialdehyde (MDA) content in whole-cell extract [40,71] with minor modifications. 2 × 10^9^ cells were washed twice with H_2_O, homogenized with 0.1% (*w*/*v*) trichloroacetic acid (TCA, Merck, Darmstadt, Germany) and broken down with glass beads. The samples were centrifuged at 10,000× *g* rpm for 5 min, and 150 µl of the supernatant were mixed with 0.8 mL of 20% TCA containing 0.5% of 2-thiobarbituric acid (Merck, Darmstadt, Germany) and heated for 30 min at 95 °C. The mixtures were immediately transferred to an ice bath and then centrifuged at 10,000× *g* for 15 min. Based on the absorbance recorded at 532 nm, MDA concentration was calculated according the standard curve obtained using malondialdehyde (0–250 µl). The fold change in the MDA content with respect to wild-type was calculated.

The intracellular lipid droplets were detected using the fluorescent lipophilic dye Nile Red (9-diethylamino-5*H*-benzo[α]phenoxazine-5-one 3 Sigma-Aldrich^®^, Darmstadt, Germany) by fluorimetric analysis as previously described [33]. Wild-type and mutant strains were grown to stationary phase in SC medium supplemented with 0.6% glucose.

### 4.5. RT-qPCR

Total RNA was extracted from cells grown to early stationary phase in SC medium supplemented with 2% galactose or 0.6% glucose. For the reverse transcription, total RNA was treated with DNase I (New England Biolabs, Ipswich, MA, USA), retro-transcribed with M-MuLV Reverse Transcriptase (New England Biolabs, Ipswich, MA, USA) with oligo (dT)20 primer (Euroclone, Milan, Italy) and murine RNase inhibitor (New England Biolabs, Ipswich, MA, USA). qPCR on retro-transcribed *ACS2*, *HFA1*, *FET3*, *FTR1*, *FIT3* and, as a reference, *ACT1* mRNA was performed by using Power Sybr Green mix with ROX Reference Dye (Kapa Biosystems, Darmstadt, Germany), supplemented with appropriate primers (Supplementary Table S1) at a final concentration of 120 nM in the AB 7300 (Applied Biosystems, Foster City, CA, USA) instrument at default settings: 50 °C for 2 min, 95 °C for 10 min, 41 cycles at 95 °C for 15 s and 60 °C for 1 min, and one cycle at 95 °C for 15 s and at 60 °C for 15 s.

### 4.6. Mitochondria Isolation, Localization Experiments, and Protein Extraction

Whole-cell extract obtained after cell wall removal by Zymoliase20T digestion (Nacalai Tesque, Kyoto, Japan) and cell disruption with a glass-teflon potter homogenizer [72], was centrifuged at 12,000× *g* for 30 min to yield the mitochondrial pellet (M) and post-mitochondria supernatant (PMS) containing the cytosolic fraction. Proteinase K protection assay for sub-mitochondrial localization study was performed as previously described [33,72,73]. The different fractions were separated by 12% SDS-polyacrylamide PAGE and probed with rat anti-HA monoclonal antibody (Roche Applied Science, Penzberg, Germany, 1:5000 dilution), mouse anti-Por1 monoclonal antibody (Abcam Mitoscience, Cambridge, UK, 1:10,000 dilution) and mouse anti-Pgk1 monoclonal antibody (Abcam Mitoscience, Cambridge, UK, 1:5000 dilution). After incubation with the appropriate secondary antibodies, ECL Western blotting substrate (Clarity™, Bio-Rad, Hercules, CA, USA) was used for final detection. To determine the Cab1 steady state level and the Lat1 and Kgd2 lipoylated subunits of PDH and α-KDH, protein extraction was performed with the TCA method by chilling the cells supplemented with 120 mM NaOH, 0.5% β-mercaptoethanol, 650 μM PMSF and 25% TCA on ice. The proteins were then suspended in Laemmli sample buffer at pH 6.8. Proteins corresponding to 1.5 OD_600_ of the initial cells were loaded on 12% or 10% SDS-PAGE and electroblotted onto nitrocellulose filters that were incubated with the specific antibody: rat anti-HA, mouse anti-Pgk1, rabbit anti-LA (Abcam Mitoscience, Cambridge, UK, 1:2000 dilution) and mouse anti-Por1. Blots were incubated with the appropriate secondary antibodies depending on the methods used for detection of the final signal and ECL Western blotting substrate (Clarity™, Bio-Rad, Hercules, CA, USA) subsequently used or fluorescence directly recorded by Chemidoc MP imager (Clarity™, Bio-Rad, Hercules, CA, USA), incubating the filters with rabbit anti-LA (anti-rabbit secondary antibody, StarBright Blue520, Bio-Rad, Hercules, CA, USA, 1:7500 dilution) and mouse anti-Por1 (anti-mouse secondary antibody; StarBright Blue700, Bio-Rad, Hercules, CA, USA, 1:10,000 dilution) Signals were quantified by Image Lab software (Bio-Rad, Hercules, CA, USA), and ratios between Cab1 and Pgk1 were calculated.

### 4.7. Screening of Selleck-FDA-Approved Drug Library

The screening was performed according to Couplan et al., [44]. Mutant strain was grown in YP medium containing 2% ethanol at 28 °C with constant shaking for 48 h. 4 × 10^5^ (*cab1*^G351S^) cells were plated onto 120 × 120 mm square Petri dishes containing 90 mL of YP solid medium supplemented with 2% ethanol. Thirty-two 6 mm sterile filters were then put on the plate and soaked with 2.5 µl of each compound of the Sellek chemical library purchased from Selleck Chemicals (Houston, TX, USA). As positive control a central spot of Δ*cab1*/pFL39*CAB1* was used while DMSO (dimethyl sulfoxide) was used as negative control. Plates were incubated at 36.5 °C and the growth was monitored for 7 days. Positive drugs were identified by a halo of enhanced growth around a filter.

The secondary screening was performed on positive molecules in the same conditions of the primary screening with the exception that 4 disks were placed on the plate, one of which soaked with DMSO.

For further analyses 5,7 dichloro-8 hydroxyquinoline (CQ_CL_) and nalidixic acid (nalH) were diluted in DMSO at concentrations ranging from 5 to 100 mM depending on their solubility and added to the growth medium at the different concentrations as indicated in the ‘Results’.

### 4.8. Statistical Analyses

All numerical data are expressed as mean ± SD, as indicated. In the figure legend, number of biologically distinct samples (independent clones analyzed) x number of technical replicates (repeated measurements of the same sample) is indicated as *n*, while the number of independent experiments performed is indicated as *N*. For statistical analyses, one-way analysis of variance (ANOVA) followed by a Bonferroni’s post hoc test was used. P-values below 0.05 were considered statistically significant.

## Figures and Tables

**Table 1:** List of the missense mutations studied. For each mutation we indicated the ability (+/−) to complement the lethal phenotype of ∆*cab1* and their effect on respiratory function. The age of onset (early/late) and the corresponding reference are also indicated.

Domain	hPANK2	yCab1	Viability +/−	Functional Effects	Age Onset Early/Late
ATP binding	D217G	D24G	−	null	E [49]
	G219V	G26V	−	null	E [50]
	G521R	G311R	−	null	E [4]
Dimerization domain	L413P	L179P	−	null	E [4]
	D447N	D213N	−	null	E [51]
	D447E	D213E	−	null	E [49]
	A509V	A299V	−	null	E [51]
	S471N	S237N	+	severe	E [4]
	I497T	I287T	+	severe	E [4]
	N500I	N290I	+	severe	E [4]
	I501I	I291T	+	severe	L [51]
	I504V	I294V	+	mild	L [52]
Protein interior	A562T	A352T	+	mild	https://www.ncbi.nlm.nih.gov/clinvar/variation/338365/ (accessed on 29 December 2020)
Surface	N404I	N170I	+	wt	L [51]
	D378G	D144G	+	wt	L [52]

## Data Availability

The data presented in this study are available in request from the corresponding author.

## Supplementary Materials

Supplementary material can be found at https://www.mdpi.com/1422-0067/22/1/293/s1.
