# Supplementary material for: Exploring Yeast as a Study Model of Pantothenate Kinase-Associated Neurodegeneration and for the Identification of Therapeutic Compounds
Source: Int J Mol Sci. 2020 Dec 30;22(1):293. doi: 10.3390/ijms22010293 (PMC7795310; doi:10.3390/ijms22010293)
Supplement: Supplementary file 1 [file ijms-22-00293-s001.zip › ijms-1036182-supplementary final/Supplementary Figure 29.12.docx]

**Figure S1**. Alignment of the Cab1 and PANK2 proteins. In green are represented the missense variants conserved between the two proteins, in purple the variants not conserved and in yellow variants not present in the yeast sequence. The alignment was obtained with ClustalW (http://www.ebi.ac.uk/Tools/clustalw).

**Table S1.** Mapping of the mutated residues conserved between human and yeast proteins. Crystal structure of PANK2 (PDB: 5e26) was loaded into PyMol. The residues were classified based on the localization.

**Figure S2.** The evaluation of optimal working concentration of CQ_CL_ and nalH was performed by measuring cell yield (OD_600_/ml) in liquid ethanol medium in the presence of the molecules. Values are means ± standard deviation. N=1; n=4 for each strain and condition. *: p<0.05; ** p<0.01 relative to *CAB1* wild-type strain grown under the same conditions of mutant strain.

**Figure S3.** (a) NADH-cytochrome c oxidoreductase (NCCR), succinate dehydrogenase (SDH) and cytochrome c oxidase (COX) activities were measured in wild type and mutant strains grown at 28°C in SC medium plus 0,2% glucose and 2% galactose with or without 1µM of CQ_CL_ or 4µM of nalH. The activities were normalized to the strain transformed with the *CAB1* wild type allele. Values are means ± standard deviation. N=3; n=9 for each strain and condition. **: p<0.01 relative to *CAB1* wild-type strain and #: p<0.05; ##: p<0.01 relative to *cab1*^N290I^ mutant strain.(b) ROS content was evaluated in wild type and mutant strains grown with or without CQ_CL_/nalH. Values are means ± standard deviation. N=4; n=8 for each strain and condition. **: p<0.01 relative to *CAB1* wild-type strain and ##: p<0.01 relative to *cab1*^N290I^ mutant strain.

**Table S2.** Primers. List of primers used in this work
