# Supplementary material for: Exploring Yeast as a Study Model of Pantothenate Kinase-Associated Neurodegeneration and for the Identification of Therapeutic Compounds
Source: Int J Mol Sci. 2020 Dec 30;22(1):293. doi: 10.3390/ijms22010293 (PMC7795310; doi:10.3390/ijms22010293)
Supplement: Supplementary file 1 [file ijms-22-00293-s001.zip › ijms-1036182-supplementary final/Table S1.docx]

| Domain | hPANK2 |
| --- | --- |
| ATP BINDING | D217 |
|  | G219 |
|  | V226 |
|  | G521 |
| DIMERIZATION DOMAIN | L413 |
|  | L424 |
|  | D442 |
|  | D447 |
|  | D452 |
|  | A469 |
|  | S471 |
|  | L494 |
|  | I497 |
|  | N500 |
|  | I501 |
|  | I504 |
|  | A509 |
| PROTEIN INTERIOR | F519 |
|  | F550 |
|  | E554 |
|  | G555 |
|  | A562 |
| SURFACE | E241 |
|  | Y361 |
|  | D378 |
|  | Y383 |
|  | N404 |
|  | Y536 |
|  | G544 |

Table S1
