# Supplementary material for: Exploring Yeast as a Study Model of Pantothenate Kinase-Associated Neurodegeneration and for the Identification of Therapeutic Compounds
Source: Int J Mol Sci. 2020 Dec 30;22(1):293. doi: 10.3390/ijms22010293 (PMC7795310; doi:10.3390/ijms22010293)
Supplement: Supplementary file 1 [file ijms-22-00293-s001.zip › ijms-1036182-supplementary final/Table S2.docx]

| **Oligo** | **Sequence** | **Use** |
| --- | --- | --- |
| CAB1FwHindIII | CAGGCAAAAAGCTTTACACGTG | Cloning |
| CAB1RvBamHI | CCCCGGATCCCACAGACATTGAAGC | Cloning |
| CAB1FwKan | GTGCATGCGAAAAGAATCGCAATGCCGCGAATTACTCAAGAGATATCCGTACGCTGCAGGTCGAC | Gene distruption |
| CAB1RvKan | GCCAAAAAAAAAGTGAAATCTATCTACGTACTTGTTTTCTTAGTAGATGATCGATGAATTCGAGCTCG | Gene distruption |
| CAB1HATerFw | TACCCATACGATGTTCCAGATTACGTCACGTAGATAGATTTCACTTTTTTT | HA tag insertion |
| CAB1HARv | AATCTATCTACGTAGCGTAATCTGGAACATCGTATGGGTAACTTGTTTTCTTAGTAGATGA | HA tag insertion |
| CAB1D24G | Fw: CTTTCAACCTTGCTATTGGAATAGGAGGCACTCTGGCTAAAGTAG  Rv: CTACTTTAGCCAGAGTGCCTCCTATTCCAATAGCAAGGTTGAAAG | Mutagensis |
| CAB1G26V | Fw: CCTTGCTATTGATATCGTTGGTACTCTGGCTAAAGTAGTCTTCTC  Rv: GAGAAGACTACTTTAGCCAGAGTACCAACGATATCAATAGCAAGG | Mutagensis |
| CAB1D144G | Fw: CCCACCAGTTCCGGCACCATGGGCTCAAAGGCTATCTACC  Rv: GGTAGATAGCCTTTGAGCCCATGGTGCCGGAACTGGTGGG | Mutagensis |
| CAB1N170I | Fw: CAATATTAAAAGTCACCGAACCAAACATTTTCAGTAGAGTAGGCG  Rv: CGCCTACTCTACTGAAAATGTTTGGTTCGGTGACTTTTAATATTG | Mutagensis |
| CAB1L179P | Fw: GTAGAGTAGGCGGTTCTTCACCTGGAGGGGAACTCTTTGGGG  Rw: CCCCAAAGAGTTCCTCCTCCAGGTGAAGAACCGCCTACTCTAC | Mutagensis |
| CAB1D213E | Fw:GGGTGACAATTCTAGCGTTGAGATGCTAGTTGGAGATATTTATGG  Rv: CCATAAATATCTCCAACTAGCATCTCAACGCTAGAATTGTCACCC | Mutagensis |
| CAB1D213N | Fw: GGGTGACAATTCTAGCGTTAACATGCTAGTTGGAGATATTTATGG  Rv: CCATAAATATCTCCAACTAGCATGTTAACGCTAGAATTGTCACCC | Mutagensis |
| CAB1S237N | Fw: GTCTAAAGTCGTCAGCTATTGCAAGTAACTTTGGTAAAGTTTTCC  Rv: GGAAAACTTTACCAAAGTTACTTGCAATAGCTGACGACTTTAGAC | Mutagensis |
| CAB1I287T | Fw: GTAAAAGTCTTCTATTCGCCACGTCCAACAATATTGGGCAAATAGC  Rv: GCTATTTGCCCAATATTGTTGGACGTGGCGAATAGAAGACTTTTAC | Mutagensis |
| CAB1N290I | Fw: CTATTCGCCATCTCCAACATTATTGGGCAAATAGCTTATTTGC  Rv: GCAAATAAGCTATTTGCCCAATAATGTTGGAGATGGCGAATAG | Mutagensis |
| CAB1I291T | Fw: GTCTTCTATTCGCCATCTCCAACAATACCGGGCAAATAGC  Rv: GCTATTTGCCCGGTATTGTTGGAGATGGCGAATAGAAGAC | Mutagensis |
| CAB1I294V | Fw: CGCCATCTCCAACAATATTGGGCAAGTCGCTTATTTGCAAGC  Rv: GCTTGCAAATAAGCGACTTGCCCAATATTGTTGGAGATGGCG | Mutagensis |
| CAB1A299V | Fw: GCTTATTTGCAAGTCAAAATCAATAATATACAGAATATATACTTTGG  Rv: CCAAAGTATATATTCTGTATATTATTGATTTTGACTTGCAAATAAGC | Mutagensis |
| CAB1G311R | Fw: TACAGAATATATACTTTGGCAGATCTTATACCAGAGGACATT  Rv: AATGTCCTCTGGTATAAGATCTGCCAAAGTATATATTCTGTA | Mutagensis |
| CAB1G351S | Fw: GAAGGCTATTTGGGTGCAATGTCTGCTTTCCTAAGCGCGTCTCG  Rv: CGAGACGCGCTTAGGAAAGCAGACATTGCACCCAAATAGCCTTC | Mutagensis |
| CAB1A352T | Fw: GGCTATTTGGGTGCAATGGGTACCTTCCTAAGCGCGTCTCGTC  Rv: GACGAGACGCGCTTAGGAAGGTACCCATTGCACCCAAATAFCC | Mutagensis |
| ACS2q | Fw: CTGCTGTTGTCGGTATTCCA  Rv: TGTGTTCTGCATCACCTTCA | qPCR |
| HFA1q | Fw: CGTGGGTGTTATTGCGGTAG  Rv: GATACCACACCTGTCCTGCT | qPCR |
| FET3q | Fw: CTACGGTTCAAACACGCACA  Rv: GGTTTGGAAAGCGTGACCAT | qPCR |
| FTR1q | Fw: AAGCAGGAGTTGACGGAAGA  Rv: TGGTAGTTTGCTCGGGAAGT | qPCR |
| FIT3q | Fw: GCTACATCCTCTAGCACCGC  Rv: GCACCCATCAAACCAGTACC | qPCR |
| ACT1q | Fw: GTATGTGTAAAGCCGGTTTTG  Rv: CATGATACCTTGGTGTCTTGG | qPCR |
| M13Fw | AGGGTTTTCCCAGTCACGACGTT | Sequencing |
| M13Rv | GAGCGGATAACAATTTCACACAGG | Sequencing |

Table S2
