# Supplementary figures and images for: Exploring Yeast as a Study Model of Pantothenate Kinase-Associated Neurodegeneration and for the Identification of Therapeutic Compounds
Source: Int J Mol Sci. 2020 Dec 30;22(1):293. doi: 10.3390/ijms22010293 (PMC7795310; doi:10.3390/ijms22010293)

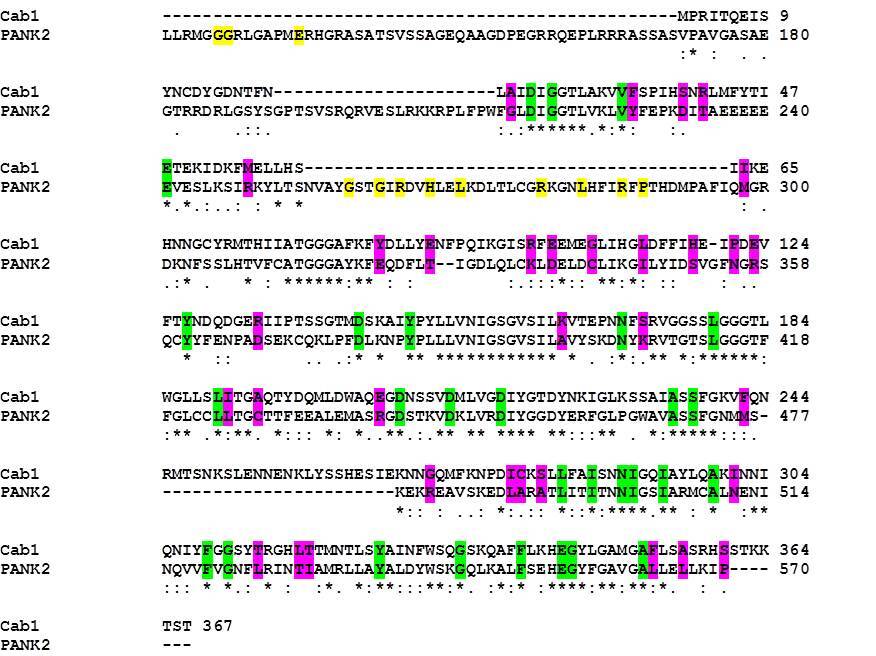

Supplement: Supplementary file 1 [file ijms-22-00293-s001.zip › ijms-1036182-supplementary final/Figure S1 300dpi.jpg]

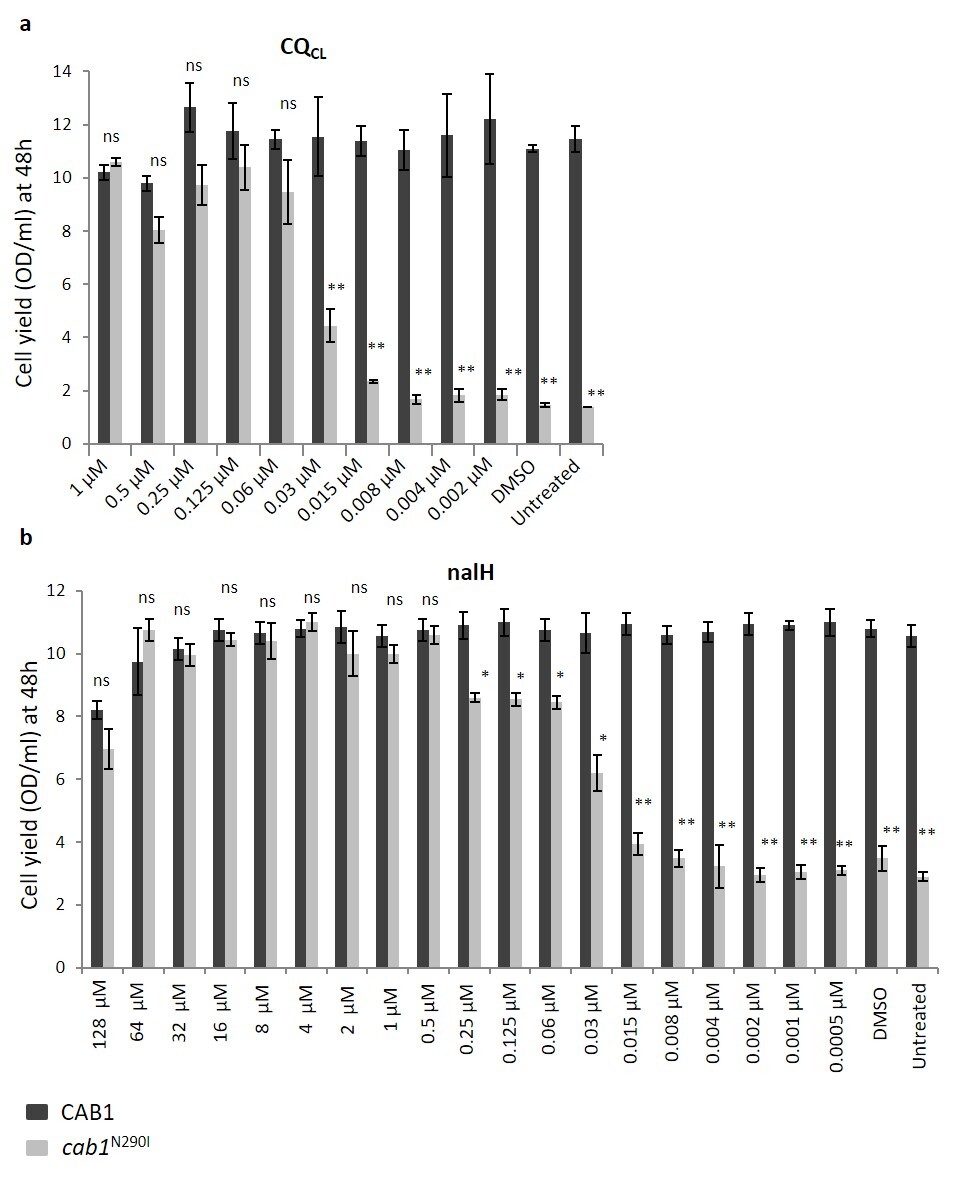

Supplement: Supplementary file 1 [file ijms-22-00293-s001.zip › ijms-1036182-supplementary final/Figure S2. 28.12.jpg]

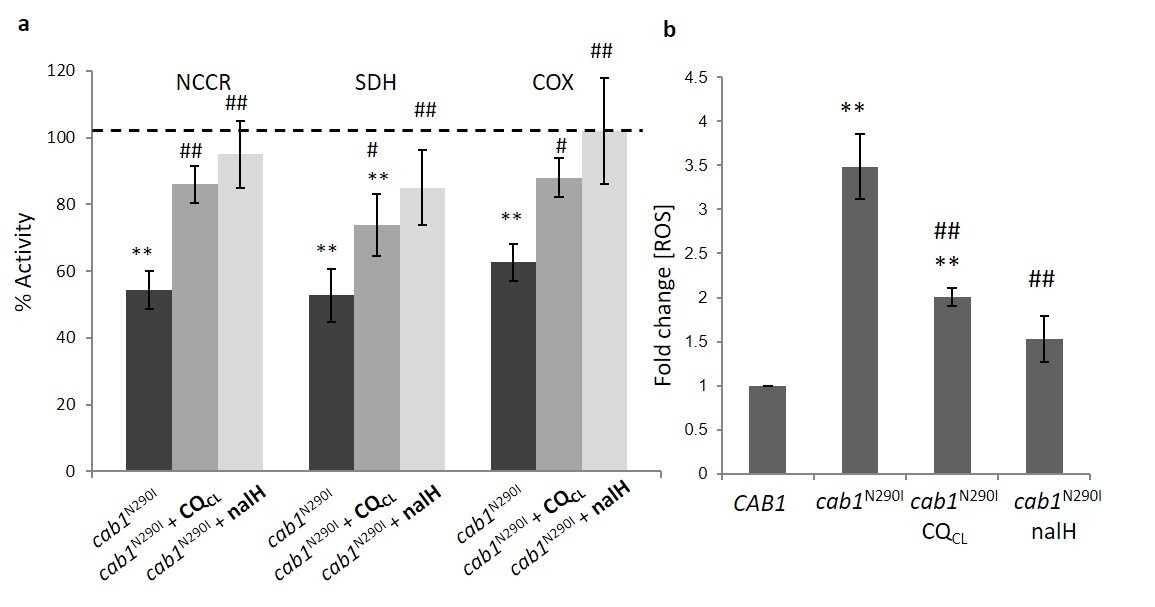

Supplement: Supplementary file 1 [file ijms-22-00293-s001.zip › ijms-1036182-supplementary final/Figure S3. 28.12.jpg]
